# Optimized virtual optical waveguides enhance light throughput in scattering media

**DOI:** 10.1038/s41467-023-40864-z

**Published:** 2023-09-14

**Authors:** Adithya Pediredla, Matteo Giuseppe Scopelliti, Srinivasa Narasimhan, Maysamreza Chamanzar, Ioannis Gkioulekas

**Affiliations:** 1 Carnegie Mellon University, Pittsburgh, PA USA; 2 Dartmouth College, United States of America (work done during Pediredla’s time at CMU), Hanover, New Hampshire USA

**Keywords:** Optogenetics, Imaging and sensing

## Abstract

Ultrasonically-sculpted gradient-index optical waveguides enable non-invasive light confinement inside scattering media. The confinement level strongly depends on ultrasound parameters (e.g., amplitude, frequency), and medium optical properties (e.g., extinction coefficient). We develop a physically-accurate simulator, and use it to quantify these dependencies for a radially-symmetric virtual optical waveguide. Our analysis provides insights for optimizing virtual optical waveguides for given applications. We leverage these insights to configure virtual optical waveguides that improve light confinement fourfold compared to previous configurations at five mean free paths. We show that virtual optical waveguides enhance light throughput by 50% compared to an ideal external lens, in a medium with bladder-like optical properties at one transport mean free path. We corroborate these simulation findings with real experiments: we demonstrate, for the first time, that virtual optical waveguides recycle scattered light, and enhance light throughput by 15% compared to an external lens at five transport mean free paths.

## Abstract (web summary)

Virtual optical waveguide can be potentially utilised in variety of applications that require in situ light steering yet the efficacy is still unclear. Here, the authors show that ultrasonically-sculpted virtual gradient-index waveguides are effective in guiding and confining light inside tissue and other scattering media, and significantly outperform external lenses at this task.

## Introduction

Chamanzar et al.^1^ recently demonstrated that ultrasound waves can be used to form virtual optical waveguides in situ in transparent or scattering compressible media, such as water or tissue. These virtual optical waveguides are sculpted non-invasively in a target medium, without inserting a physical optical waveguide or lens. In particular, as ultrasonic pressure waves are applied to the medium, they modulate its local refractive index, creating continuously-varying refractive index profiles analogous to those in gradient-index (GRIN) optical waveguides. Depending on the sculpted profile, the resulting virtual optical waveguides can steer light to produce focused spots^1^ and complex spatio-temporal patterns^2^ inside the medium, or relay images through the medium^3^.

Virtual optical waveguides can be implemented using ultrasonic waves from phased arrays^1^, transient ultrasonic waves^4^, and laser-induced nonlinear acoustic waves^5^. These implementations can be useful in a variety of applications that require in situ light steering. For example, virtual optical waveguides can potentially be used in biological applications, to enable light delivery and confinement through absorptive and scattering biological tissue for optogenetic stimulation of cells, fluorescence imaging, calcium imaging, photodynamic and photothermal therapy^6–11^. More generally, virtual optical waveguides can improve the performance and flexibility of the methods and processes for light manipulation in clear and turbid compressible media^3^.

Motivated by this strong potential for applications, our goal in this paper is to systematically analyze and characterize the effectiveness of virtual optical waveguides for guiding light inside scattering media, and to derive principles for configuring virtual optical waveguides. To this end, we emphasize a specific use case of virtual optical waveguides, namely, confining light inside a medium from the beam incident to it. Within this context, we set out to answer three related questions: First, how is the ability to confine light affected by ultrasonic parameters (e.g., amplitude, frequency) and optical properties of the medium (e.g., extinction coefficient). Second, how does the ability to focus light differ between transparent media—where there is only ballistic light—and turbid media—where there is both ballistic and scattered light. Third, how does confining light inside the medium using virtual optical waveguides compare to focusing light using an external lens. We investigate these questions for both media relevant for biological applications (i.e., tissue), and more general media that may be relevant for other applications (e.g., imaging in murky water).

Answering these questions by relying exclusively on lab experiments would be impossible, because of both the impractically large number of required experiments, and the complete lack of physical hardware for certain types of measurements (e.g., separately measuring light paths that scatter a different number of times inside a turbid medium). Instead, we adopt a hybrid approach. First, we develop a simulator of light traveling inside turbid and transparent media with sculpted virtual optical waveguides (in methods). Our simulator accounts for both the multiple scattering and continuous refraction effects that light undergoes under these conditions, by using Monte Carlo rendering techniques to solve the refractive radiative transfer equation (RRTE)^12^. Additionally, our specialized simulator is two orders of magnitude faster than previous general-purpose simulators of the RRTE^13^, without sacrificing accuracy. Second, we use our simulator to thoroughly quantify the light confining performance of virtual optical waveguides, and compare it with focusing using external lenses. We perform simulations for two different ranges of ultrasound parameters and for different material properties: (i) media and ultrasonic settings motivated by prior work^1^ that help better understand the interplay between ballistic and scattered light confined and guided through the virtual optical waveguides; and (ii) tissue-like media under ultrasonic settings satisfying safety constraints. Third, we validate our simulation-based analysis, by performing real experiments and comparing their results with simulation predictions.

We summarize the findings of our simulations and experiments as follows: There are multiple configurations of ultrasonic amplitude (refractive index contrast) and frequency parameters that focus an incident beam to a point inside a medium. These configurations perform differently in terms of light throughput—-the amount of light they successfully guide to a small area around the focus point. Additionally, the configurations maximizing light throughput are different for transparent and turbid media, demonstrating the need to consider both ballistic and multiply-scattered light when configuring virtual optical waveguides. From our quantitative analysis, we draw the following conclusions on the light throughput performance of virtual optical waveguides: (1) ultrasound parameters that result in focal configurations can significantly enhance light throughput; (2) higher-order focal configurations result in better scattered light throughput performance; and (3) within focal configurations of the same order, lower frequencies (and hence, higher amplitude) result in better performance. We show that these conclusions can be explained using physics principles, and thus correspond to generally-applicable insights that can help configure virtual optical waveguides with improved performance in other application settings.

To demonstrate this, we use these insights to configure virtual optical waveguides for settings suitable for biological applications—media with optical properties similar to human bladder, ultrasound parameters satisfying safety constraints. We show in simulation that our virtual optical waveguide configurations improve light throughput by 300% compared to configurations derived using the previous design principles based on only ballistic light paths^1^ at depth of 5 mean free paths; and by 50% compared to an ideal external lens at depth of one transport mean free path. Additionally, we use our insights to configure virtual optical waveguides that, in real experiments, improve light throughput by 15% compared to a real external lens at depth of 5 transport mean free paths. Our simulation and experimental results highlight that carefully configured virtual optical waveguides improve light throughput performance compared to external lenses, thanks to the waveguides’ ability to not only focus ballistic light, but also recycle and guide scattered light.

## Results

### Technical background

**Fig. 1:**
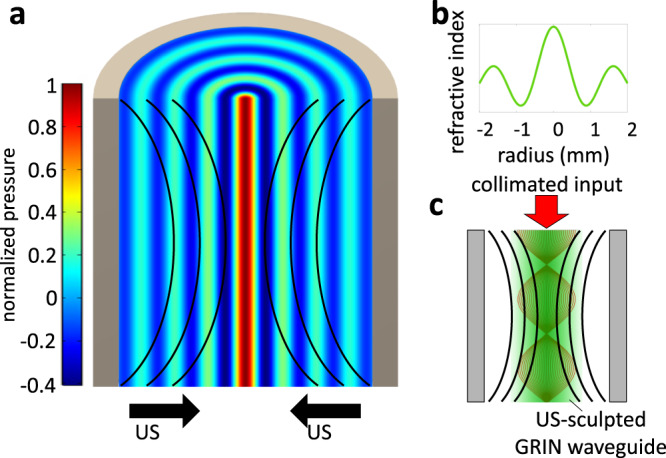
Physics of ultrasonically sculpted virtual optical waveguide. **a** Ultrasound generates a local standing wave within the cavity of a cylindrical transducer immersed in water or a scattering medium. **b** Pressure modulates the medium and sculpts a refractive index profile. **c** The refractive index behaves like a GRIN waveguide, confining and focusing an input beam of light within the cavity of the transducer. Figure adapted from Scopelliti et al.^38^.

We first detail the technical background required for understanding the virtual optical waveguideĊhamanzar et al.^1^ demonstrated that ultrasound can be used to sculpt a gradient-index (GRIN) virtual optical waveguide into transparent and scattering media. They performed proof-of-concept demonstrations by using a cylindrical ultrasonic transducer to induce standing pressure waves into the medium (Fig. 1a). These pressure waves modulate its local density and, consequently, refractive index according to the Lorentz-Lorenz relationship^14^ (Fig. 1b).

Figure 1 shows the pressure pattern and resulting refractive index profile within the cylindrical geometry. The radial profile of the refractive index can be described as^2, 15^

1 $$n\left(r,\phi,t\right)={n}_{o}+{{\Delta }}n{J}_{0}\left({k}_{r}r\right)\sin \left(2\pi ft\right),$$

where: *r* and *ϕ* are radial and azimuthal coordinates; *t* is time; *n*_*o*_ is the background refractive index of the medium; Δ*n* is the refractive index contrast, equal to the difference between the maximum amplitude of the refractive index and *n*_*o*_, which is approximately proportional to ultrasonic amplitude; *J*_0_ is the 0th-order Bessel function of the first kind; *k*_*r*_ = 2*πf*/*V*_us_ is the radial component of the ultrasonic wavevector; *f* is the ultrasonic frequency; and *V*_us_ is the velocity of ultrasound in the medium.

The sculpted GRIN optical waveguide confines and focuses light within the high refractive index region (Fig. 1c), similar to a conventional GRIN waveguide that focuses ballistic light. In this paper, we are looking to quantitatively and qualitatively characterize this ability of virtual optical waveguides to focus light inside media. With the analogy with a GRIN waveguide in mind, we quantify the confinement ability of a virtual optical waveguide by considering its light throughput, which is defined as the light flux reaching a target area around a specified focus point inside the medium. We will be using light throughput as the performance metric to compare how sculpting a virtual optical waveguide improves light confinement within the target medium relative to the light throughput in an unmodulated medium, and also in comparison with alternative focusing techniques such as using an external lens.

**Table 1:** In this paper, we characterize the effect of various parameters including ultrasound and scattering medium parameters

type	Parameter	Default value
setup	beam diameter	500 μm
	beam intensity	75000 a.u.
	target region radius (*r*)	50–100 μm
	medium length (*L*)	30 mm
ultrasonic	amplitude (Δ*n*)	0.0001–0.05
	frequency (*f*)	0.3–1.5 MHz
	speed (*V*_*us*_)	0.0001–0.05
medium	base refractive index (*n*_0_)	1.3333
	albedo (a)	0.99
	extinction coefficient (*σ*_*t*_)	0–166.7 m^−1^
	phase function anisotropy (*g*)	0.9

**Fig. 2:**
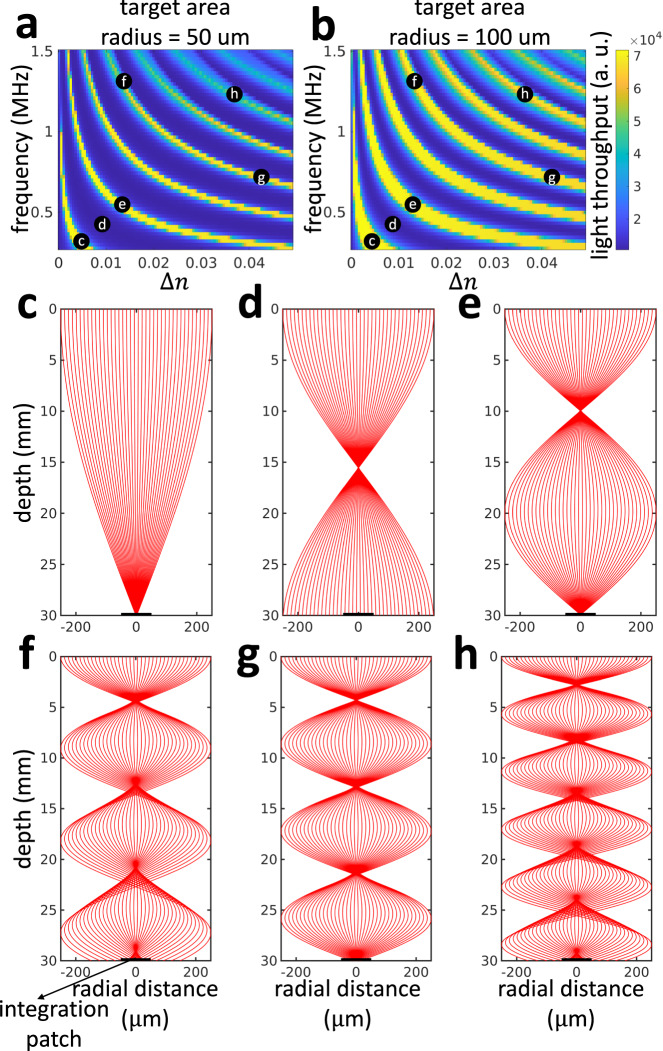
Ray diagrams are enough to understand the behavior of light in a transparent medium (simulation). In the absence of scattering, the behavior of virtual optical waveguides can be entirely described by ray diagrams. From (**a**, **b**), we can observe that the locus of high-intensity frequency-amplitude combinations are approximately on a hyperbola, and decreasing the radius of the integration patch makes the hyperbolas thinner. The high-intensity frequency-amplitude combinations are focal combinations where the photons are focused on the target plane (**c**, **e**). If the photons are defocused, the intensity will be low (**d**). Within a given focal locus, high-frequency configurations (**f**) will lead to higher aberrations than high-amplitude configurations (**g**). Higher-order focal loci will cause higher aberrations (**h**), and hence ray diagrams suggest that we need to operate at first focus.

**Fig. 3:**
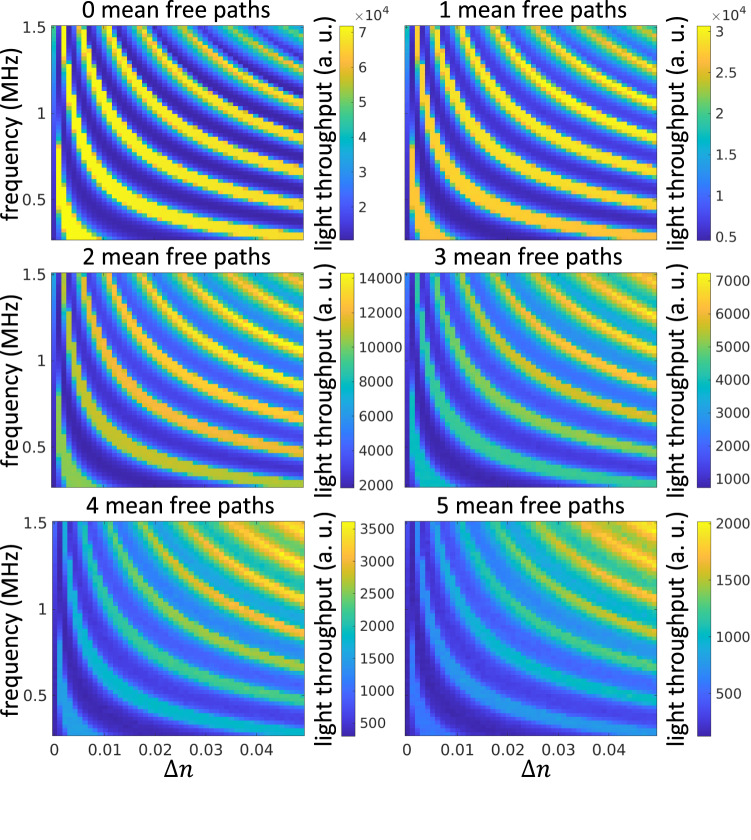
Effect of varying ultrasonic amplitude vs. frequency at different scattering levels (simulation, target region of radius *r* = 100 μm). We observe that the loci of high light-throughput frequency-amplitude pairs are approximately on a set of hyperbolas. Each of these hyperbolas corresponds to a focal locus of the waveguide. As the amount of scattering increases, the maximum light throughput decreases (note the colorbar scales), and the optimal frequency-amplitude pairs occur at higher-order focal loci. Note that we set the beam flux to 7.5 × 10^4^a.u. throughout the paper, and that peak power for even 0 MFP is <7.5 × 10^4^a.u. due to Fresnel reflection at the boundaries.

**Fig. 4:**
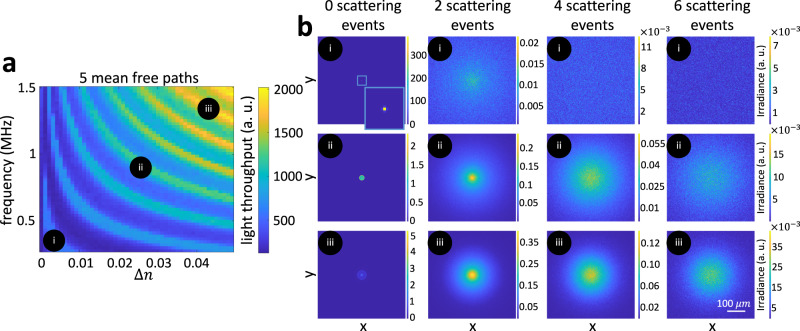
Scattering event analysis demonstrates that higher-order focal locus better guides scattered photons (simulation, target region of radius *r* = 100 μm). **a** frequency-amplitude map for a 5MFP sample with three frequency-amplitude configurations highlighted at (i) low-order focus, (ii) intermediate-order focus, and (iii) higher-order focus. **b** We decompose all photons at these three configurations based on the number of scattering events and show simulated images for zero, two, four, and six scattering events. 0 scattering events shows ballistic photons only, and we observe that lower order focuses ballistic photons better, whereas higher-order focuses suffers from aberrations. As the number of scattering events increases, the photons are less confined as visible from both the spatial spread of the intensity image and the color bar. Compared to the lower order focus, the higher-order focus better confines the scattered photons, and hence, higher-order focus better guides scattered photons.

**Fig. 5:**
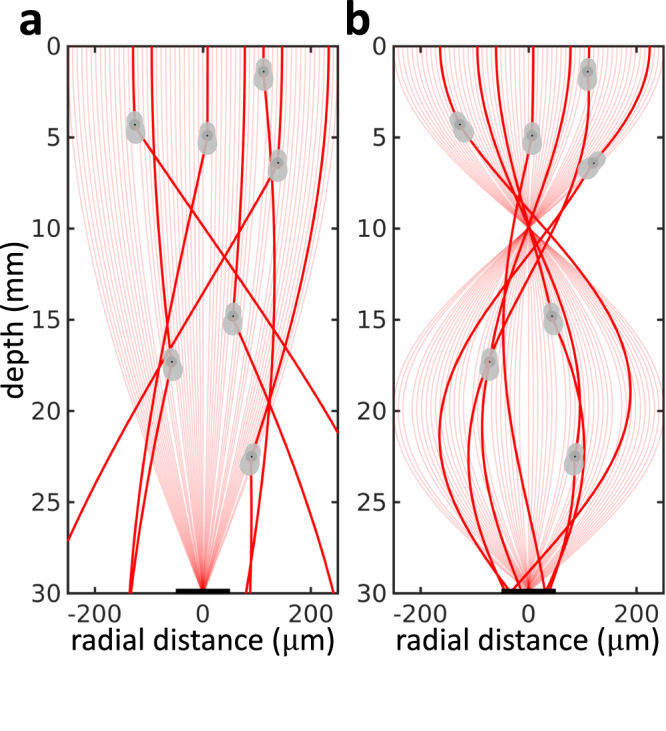
Scattered photon trajectories demonstrate that higher-order focus better guides scattered photons (simulation). We plot a few scattered photon trajectories (thick red lines) for (**a**) first focus and (**b**) second focus. The distribution of the position of the scattered photons depends on the medium’s scattering length, and the distribution of the scattering directions depends on the phase function. This illustration shows a few scattered photon trajectories sampled randomly, as showing all possible trajectories will make the image cluttered. The trajectories of the first focus photons appear to be straight due to the small refractive index gradient. The scattered photons are better guided to the target plane with the second focus compared to the first focus suggesting that higher-order focus better guide scattered photons.

In this table, we show the typical values used in this paper (Figs. 2–5 and 9).

We are particularly interested in light throughput comparisons in turbid media, which scatter (potentially multiple times) some of the light traveling through them. External lenses can focus ballistic (i.e., unscattered) light, but have no control over the scattered light, which will deviate from its original trajectory and thus not arrive at the target area. By contrast, the in situ virtual optical waveguides can gradually reroute scattered light towards the target area of interest. As a result, some portion of the scattered light that would be otherwise dispersed in the medium is “recycled” and guided towards the target area, resulting in increased light throughput. This increased light throughput is important for light-based biological applications, including photodynamic or photothermal therapy, and optogenetic stimulation^6–11^, where increasing light throughput at a certain region can be more important than achieving high spatial resolution (e.g., for single-cell-resolution imaging).

In the rest of the paper, we analyze how the light throughput of a virtual optical waveguide varies as a function of two sets of parameters. The first is the ultrasound parameters that control the sculpted refractive index profile of the virtual optical waveguide. These include the ultrasonic frequency and ultrasonic amplitude, which determines the refractive index contrast Δ*n*. For any given values of these parameters, we additionally consider how light throughput depends on the medium, and in particular, its optical properties (extinction coefficient).

The need to consider both ultrasonic and medium parameters in conjunction creates a very high-dimensional parameter space that is impractical to characterize using only lab experiments. There is no closed-form expression for computing light throughput analytically in turbid media, which makes analysis even more complex. We discuss a rigorous numerical analysis using a computationally-efficient and physically-accurate simulator, based on Monte Carlo rendering, of light propagation in turbid media modulated by ultrasound. We discuss details about the simulator in the Methods section.

### Characterization of the parameter space

In the Methods section, we built a simulator for virtual optical waveguides. In this subsection, we use our simulator to understand the effect of ultrasonic parameters and medium properties on light throughput. To this end, we perform simulations for different values of ultrasonic amplitude, ultrasonic frequency, and medium extinction coefficient. We perform simulations in both transparent and turbid media, to highlight the qualitatively different effects of ultrasonic parameters on light throughput for the two cases. We additionally vary the size of the target region, using disks of radius *r* = 50 μm and *r* = 100 μm. We list all the parameters of the simulated system in Table 1. We choose these parameters to match the waveguide length and the medium used by the most closely-related prior work (Chamanzar et al.^1^); and because they help demonstrate the trade-off between guided and aberrated light that we discuss later in this subsection. We will later leverage these insights to analyze a different set of parameters better suited to biological applications; we will show how our findings from this subsection can facilitate analysis of that parameter set.

We will first perform simulations in transparent media. Figure 2a, b shows light throughput simulations for a transparent medium (i.e., optical depth 0 MFP), for target regions of radii of *r* = 50 μm (Fig. 2a) and 100 μm (Fig. 2b). We plot light throughput as a function of the refractive index contrast Δ*n* (horizontal axis), which is proportional to the ultrasonic amplitude, and the ultrasonic frequency *f* (vertical axis).

In both cases, we observe that light throughput takes large values along approximately hyperbolic contours. To understand this behavior, in Fig. 2c–h, we use ray tracing diagrams to visualize the light trajectories through the virtual optical waveguide for a few different Δ*n*-*f* configurations from Fig. 2a, b. We first observe that configurations with high throughput (c, e, f, g, h) produce virtual optical waveguides that focus all light on the target region. We refer to configurations of this kind as focal configurations. By contrast, configurations with low throughput (d) produce virtual optical waveguides that are out of focus on the target region, and thus lose a lot of light.

When we compare high throughput configurations from different contours (i.e., c versus e, f, g, and h), we observe that each contour corresponds to virtual optical waveguides that have different numbers of intermediate focus points before focusing on the target region. This corresponds to the beating effect of GRIN waveguides. We use the term *k*^th^-order focal locus to refer to the contour of focal configurations where the target region is the *k*^th^ focus point.

To understand the hyperbolic shape of focal loci, we note first from Hamilton’s Eq. (7) that the bending of light rays in the virtual optical waveguide is determined by the spatial gradient ∇ *n*(**x**) of the waveguide’s refractive index. From Eq. (1), this gradient equals:

2 $$\frac{\partial n\left(r,\phi,t\right)}{\partial r}={k}_{r}{{\Delta }}n{J}_{0}^{{\prime} }\left({k}_{r}r\right)\sin \left(2\pi ft\right),$$

We observe that the refractive index gradient changes approximately proportionally to ultrasonic amplitude (through Δ*n*) and ultrasonic frequency (through *k*_*r*_). Therefore, to maintain a refractive index gradient that results in focusing, an increase in the ultrasonic amplitude needs to be counteracted by an approximately inversely-proportional decrease in ultrasonic frequency, and vice versa. Consequently, the focal loci have approximately hyperbolic shape.

To understand light throughput behavior within each focal locus, from the ray diagrams in Fig. 2f, g, we observe that focusing aberrations are more prominent at higher frequencies than at higher amplitudes within a given focal locus. If the target region is not large enough to capture all the aberrated rays, the light throughput will be smaller at high frequencies than at high amplitudes. We indeed observe this behavior in Fig. 2a (target region of radius *r* = 50 μm). By contrast, this behavior is less pronounced for larger target regions that capture the aberrated rays, as we see in Fig. 2b (target region of radius *r* = 100 μm).

Lastly, to understand light throughput behavior across focal loci of different orders, we observe from the ray diagrams in Fig. 2c, e, h that aberrations increase as focus order increases. Consequently, and similarly to the previous paragraph, when the target region is not large enough to capture the aberrated rays, increasing focus order will decrease light throughput (Fig. 2a, b).

We can summarize the findings of this section as follows: In transparent media, the focusing analysis for only ballistic light suggests that we can maximize light throughput using virtual optical waveguides corresponding to 1^st^-order focus and high ultrasonic amplitude-low ultrasonic frequency.

We will next perform simulations in turbid media. In Fig. 3, we show frequency-amplitude plots analogous to the ones in the previous section, but for turbid media of different optical densities. We report optical density indirectly, as the optical depth (in mean free paths) of a virtual optical waveguide of fixed geometric length *L* = 30 mm. By increasing the medium’s extinction coefficient *σ*_*t*_, we increase optical density, and thus optical depth. For all simulations in this section, we set the albedo of the medium as *a* = 0.99, and its phase function *f*_*s*_ as a Henyey-Greenstein phase function^16^ of anisotropy factor *g* = 0.9.

We observe that, when optical density increases, the ultrasonic frequency-amplitude configurations that produce high light throughput correspond to higher-order focal loci. This is contrary to the case of transparent media (i.e., optical depth 0 MFP). To understand the reason for this difference, in Fig. 4, we visualize irradiance values at the target area, separately for ballistic light and light that has scattered different numbers of times. We compare these visualizations for focal configurations of first, fourth, and seventh order. The virtual optical waveguide for the first-order focal configuration focuses ballistic light more effectively than waveguides for higher-order focal configurations. However, as the number of scattering events increases, the virtual optical waveguides for higher-order focal configurations guide scattered light better than the ones for lower-order focal configurations. We note that this type of analysis is enabled by our simulator and is not possible using traditional analytical or experimental analysis. This is because, even though it is possible to separately measure flux due to ballistic versus scattered light paths—by using techniques such as optical coherent tomography^17–19^, or time-domain diffuse optical tomography^20^—there is no imaging technology that makes it possible to separately measure flux due to light paths that have scattered only *N* > 1 number of times. From the scattering event analysis, we could also observe that virtual optical waveguides confine not just snake like photon paths (2–4 scattering events) but also multiply scattered photon paths.

Based on the analysis of Fig. 4, we can explain the change in light throughput behavior between transparent and turbid media as being due to the trade-off between aberrated ballistic light and guided scattered light. As we explained in the case of transparent media, the larger frequency and amplitude values of higher-order focal loci increase aberrations; as a result, some ballistic light rays do not reach the target region, decreasing light throughput. On the other hand, higher-order focal loci also increase guided scattered light, improving light throughput. Therefore, in the case of turbid media, the optimal ultrasonic frequency-amplitude pairs strike a balance between reducing aberrated ballistic light and increasing guided scattered light.

To provide intuition as to why higher-order focal loci increase guided scattered light, we show in Fig. 5a few single-scattered light rays, overlaid on ballistic ray diagrams for first-order and second-order focal configurations. We note that, due to the stochastic nature of light rays in turbid media, it is impossible to show all scattered rays. The virtual optical waveguide formed by the second-order focal configuration better guides the scattered rays towards the target region, compared to the one formed by the first-order focal configuration. This is due to the fact that as the refractive index gradient for the second-order focal configuration is higher, scattered light rays bend more, making it easier to mitigate scattering away from the ballistic ray.

We conclude that, through an analysis that accounts for both ballistic and scattered light, we can discover ultrasonic frequency-amplitude configurations that maximize light throughput in turbid media, by optimally balancing the trade-off between reducing aberrated ballistic light and increasing guided scattered light. By contrast, using an analysis that accounts for only ballistic light, we can arrive at sub-optimal ultrasonic configurations. For example, in Fig. 4, using the configuration (i) suggested by simulating only ballistic light leads to a 300% light throughput reduction compared to the optimal configuration (iii).

### Analysis for biological applications

In the previous subsection, we demonstrated the trade-off between the aberration of ballistic photons and the guiding effect of virtual optical waveguides on scattered light. In this subsection, we consider how to maximize light throughput enhancement, while at the same time taking into account an important safety constraint for biological tissue. This constraint forces us to consider virtual optical waveguide configurations that operate at much higher frequencies than those we analyzed in the previous subsection. Still, we can leverage the insights we gained in the previous subsection to configure virtual waveguides for enhancing light throughput in biological tissue.

In particular, the mechanical index (MI) indicates the mechanical effects of ultrasound on biological tissue. Large values of MI indicate a higher chance of cavitation and consequent tissue damage. The MI is a function of ultrasonic peak negative pressure and frequency:

3 $${{{{{{{\rm{MI}}}}}}}}=\frac{{P}_{r}}{\sqrt{f}},$$

where *P*_*r*_ is the derated peak rarefaction pressure, expressed in MPa. The Food and Drug Administration (FDA) requires MI ≤ 1.9 for diagnostic applications of ultrasound, to prevent mechanical damage to tissue^21^.

We use our simulator to analyze light throughput in human tissue of virtual optical waveguides, produced by ultrasonic frequency-amplitude configurations that comply with the MI safety limit. We focus our analysis on human bladder, where light-based methods are used to treat cancer^22–24^. Light delivered externally through the bladder wall scatters after propagating for only a few millimeters. For our analysis, we assume bladder wall depth *L* = 2.67 mm, light wavelength 630nm, and target region radius *r* = 50 μm. These values are representative of diagnostic applications (e.g., optical coherence tomography), and therapeutic intervention applications (e.g., photodynamic therapy). For the optical properties of tissue at this wavelength, we use *σ*_*t*_ = 3.75 mm^−1^, *a* = 0.992, and *g* = 0.9; these values are in the range of possible human bladder scattering properties previously reported in the literature^25^. We simulate ultrasonic frequencies up to 40 MHz, which is the range commonly employed in ultrasonic diagnostic applications. Higher ultrasonic frequencies are possible, but the acoustic propagation loss would be high (~0.3 − 0.6dB ⋅ cm^−1^ ⋅ MHz^−1^ for human tissue), limiting the maximum penetration of ultrasonic waves into the target medium^26, 27^.

Analyzing the light throughput of virtual optical waveguides in human bladder requires performing simulations for multiple ultrasonic frequency-amplitude configurations, as in Fig. 3. However, as the optical depth of bladder tissue at 630nm is as high as ~10MFP, running multiple simulations for this setting is computationally prohibitive.

**Fig. 6:**
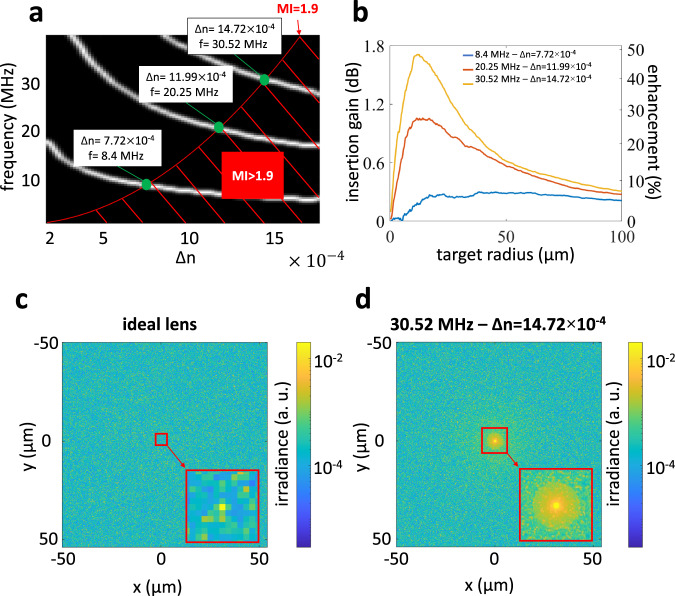
Safety of US parameters in biological applications and relative light throughput enhancement vs. ideal lens for human bladder (simulation). The bladder has geometric depth 2.67 mm and optical depth 10MFP. **a** A frequency-amplitude map, calculated using a range of frequencies commonly used in biological applications, is overlapped with the MI = 1.9 curve (in red), determining three sets of safe parameters to generate a virtual optical waveguide in human bladder tissue. **b** Insertion gain versus target radius for each of the three sets of frequency-amplitude parameters. We calculate gain relative to an external ideal lens and corresponds to a light throughput enhancement of up to 50%. 2D intensity distribution of light confined through the human bladder sample by (**c**) the lens and (**d**) the highest frequency virtual optical waveguide.

We leverage our observations from the previous subsection to constrain the parameter space we need to explore, and thus greatly reduce the computational cost. In particular, we first note that, to achieve high light throughput, we only need to simulate focal ultrasonic configurations for a medium of the same geometric depth as bladder. We show the corresponding focal loci in Fig. 6a, together with the curve for MI = 1.9 obtained using Eq. (3).

Second, we note that, within each focal locus, virtual optical waveguides for higher ultrasonic frequency have lower light throughput than those for lower ultrasonic frequency, due to increased aberrations. Therefore, at each focal locus, we need to consider only the configuration with the largest ultrasonic refractive index contrast that does not violate the MI safety limit. This reduces the frequency-amplitude configurations we need to simulate to just three, shown on Fig. 6a.

As additional validation that the ultrasonic frequency-amplitude configuration with the highest light throughput are among the three we identified, we simulate six randomly chosen configurations. We find that they all result in lower light throughput (at *r* = 50 μm) than the three candidate configurations we identified using the observations from the previous subsection.

We simulate the light throughput for these three configurations, and compare it against the light throughput when using an ideal external lens for focusing. By an ideal lens, we refer to an aberration-free lens that is not diffraction-limited (all light focuses at a single point), and does not attenuate light (non-absorptive). To simulate an ideal lens, first, we use the thin lens approximation^28^ to compute the input light field. Second, we simulate light transport through the scattering medium using the same simulator as for virtual optical waveguides simulations, but setting Δ*n* = 0. Even though such an ideal lens does not exist in reality, we use it to compare the performance of virtual optical waveguides against the most favorable scenario for external optics, and show that virtual optical waveguides offer advantages even against this scenario.

Intuitively, we expect virtual optical waveguides to improve light throughput relative to external lenses. This is because, as we showed in the previous subsection, virtual optical waveguides can not only focus ballistic light, but also recycle and guide scattered light. By contrast, external lenses can only focus ballistic light from outside the tissue, and have no control over light that scatters after penetrating the tissue. As not all recycled light will arrive at exactly the focus point, the light throughput enhancement will depend on the size of the target region.

To quantify light throughput enhancement, we perform simulations for different concentric circular target regions of increasing radii. Then, for each focusing technique, we first quantify the loss of light due to scattering in the turbid medium. For this, compute the insertion loss, equal to the relative light flux reaching the target region in a turbid medium versus a transparent medium. In dB, this becomes:

4 $${{{{{{{\rm{insertion}}}}}}}}\_{{{{{{{\rm{loss}}}}}}}}\left(r\right)\equiv -10\log \left(\frac{{F}_{s}\left(r\right)}{{F}_{t}\left(r\right)}\right),$$

where: ${F}_{t}\left(r\right)$ is the total light flux reaching the target region of radius *r* when the medium is transparent (with no absorption and scattering); and ${F}_{s}\left(r\right)$ is defined similarly for the turbid medium. We assume that the transparent medium has the refractive index of water, mimicking the average bulk refractive properties of real tissue.

We then compare the performance of virtual optical waveguides and an ideal external lens by comparing their insertion losses: As a smaller insertion loss is better, we can compute the insertion gain achieved by using a virtual optical waveguide instead of an external lens (in dB) as:

5 $${{{{{{{\rm{insertion}}}}}}}}\_{{{{{{{\rm{gain}}}}}}}}\left(r\right) \equiv \, {{{{{{{{\rm{insertion}}}}}}}}\_{{{{{{{\rm{loss}}}}}}}}}^{{{{{{{{\rm{lens}}}}}}}}}\left(r\right)\\ -{{{{{{{{\rm{insertion}}}}}}}}\_{{{{{{{\rm{loss}}}}}}}}}^{{{{{{{{\rm{US}}}}}}}}}\left(r\right).$$

The insertion gain is directly related to the relative light throughput enhancement defined as:

6 $${{{{{{{\rm{enhancement}}}}}}}}\left(r\right)\equiv \frac{\frac{{F}_{{{{{{{{\rm{s}}}}}}}}}^{{{{{{{{\rm{US}}}}}}}}}\left(r\right)}{{F}_{{{{{{{{\rm{t}}}}}}}}}^{{{{{{{{\rm{US}}}}}}}}}\left(r\right)}-\frac{{F}_{{{{{{{{\rm{s}}}}}}}}}^{{{{{{{{\rm{lens}}}}}}}}}\left(r\right)}{{F}_{{{{{{{{\rm{t}}}}}}}}}^{{{{{{{{\rm{lens}}}}}}}}}\left(r\right)}}{\frac{{F}_{{{{{{{{\rm{s}}}}}}}}}^{{{{{{{{\rm{lens}}}}}}}}}\left(r\right)}{{F}_{{{{{{{{\rm{t}}}}}}}}}^{{{{{{{{\rm{lens}}}}}}}}}\left(r\right)}}\times 100\%.$$

In the above, “US" and “lens" represent the virtual optical waveguide and ideal lens cases, respectively.

In Fig. 6b, we show the insertion gain and relative light throughput enhancement for the three ultrasonic frequency-amplitude configurations we show in Fig. 6a. We observe that, for target regions of small radii, the external lens and virtual optical waveguide have similar performance; this is because the light reaching the target region is mainly ballistic light. However, when the target region radius increases, the performance improvement from using a virtual optical waveguide becomes significant, as more and more recycled scattered light contributes to the flux at the target region. We note, in particular, that using a virtual optical waveguide can result in relative light throughput enhancement as high as 50% for a target region of radius *r* = 12 μm at the highest ultrasonic frequency (*f* = 30.52 MHz). The effect of light recycling is also visible in Fig. 6c, d, where we compare irradiance values at the target region when using an external lens versus a virtual optical waveguide. In the latter case, the aberrated ballistic focus is surrounded by a faint circular region due to recycled scattered light. This region is absent in the case of the ideal external lens, where scattered light is not recycled.

### Hardware results

So far, we have studied the effect of different ultrasound parameters on the performance of virtual optical waveguides in scattering media using simulations. In particular, we have shown that using virtual optical waveguides can significantly enhance light throughput when delivering light into scattering media using these virtual optical waveguides. In this subsection, we present experimental results that verify this light throughput enhancement. In particular, we performed experiments where we sculpted virtual optical waveguides into a scattering medium using ultrasonic waves launched into the medium by a cylindrical transducer operating at a frequency of ~7.8 MHz. The transducer we used for these experiments has a length of 11 mm, compared to the 2.67 mm transducer we simulated in the previous subsection. These choices for ultrasonic frequency and length are due to practical considerations and current availability of piezoelectric cylindrical transducers. In the future, it should be possible to design transducers with higher frequency and shorter length, in order to match the transducer parameters we obtained in the previous subsections and achieve the corresponding higher light throughput.

In our experiments, we compared the light throughput enhancement achieved by the virtual optical waveguide to the light delivered to the target region of interest by an external lens with matching spot size. The matching spot size ensures that the performance of the external lens and the virtual optical waveguide are similar for ballistic light. Our experimental results indeed confirm that the light throughput is significantly enhanced by the virtual optical waveguide, corroborating that some of the otherwise scattered and lost light is recycled and guided towards the target region of interest, as predicted by our simulation in biological applications.

**Fig. 7:**
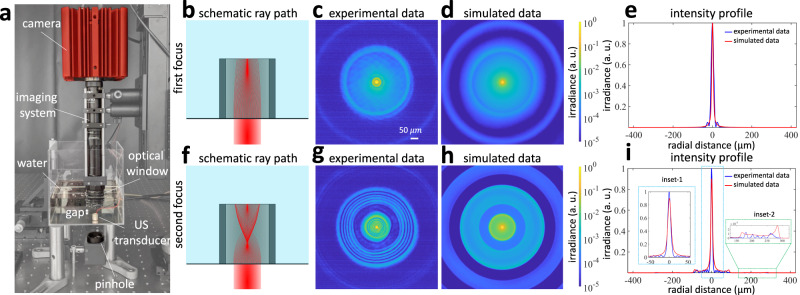
Experimental setup schematic and comparison between real data and simulated data. **a** Picture of the customized setup used in experiments. **b** Schematic ray path at 7.5V (first focus). **c** Experimental data and (**d**) simulated data for the first focus; (**e**) the cross-sectional intensity profiles show a good match. **f** Schematic ray path at 33V (second focus). **g** Experimental data and (**h**) simulated data for the second focus; (**i**) the cross-sectional intensity profiles show some minor mismatches. We exaggerated the mismatches in inset-1 and inset-2 to highlight the differences. In inset-1, the mismatch is due to the transducer (hardware). Ideally, the profile would be symmetrical. However, due to the electrical connectors, at high ultrasonic amplitude and frequency, the experimental data shows a slight asymmetry. Note that the mismatch is not large, except for the shift in the real data. In inset-2, the mismatch occurs due to unmodeled diffraction in the simulator.

**Fig. 8:**
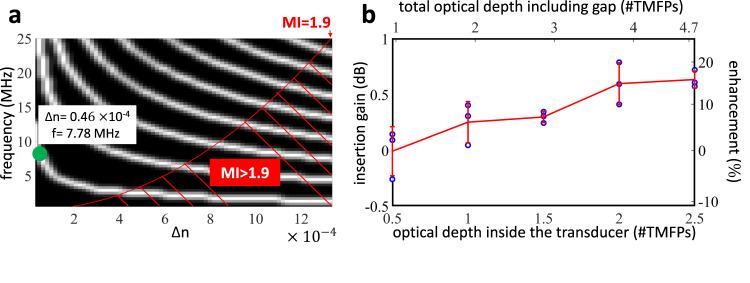
Experimental demonstration of light recycling ((a) simulation and (b) real data). **a** The transducer used in the experiments is 11 mm long, which results in more focal loci compared to Fig. 6a. We operated the transducer at a frequency of 7.8 MHz and at the first focal locus, because of constraints by the transducer’s operation regime. Designing transducers that could operate at higher amplitude or frequency would result in higher light throughput. **b** Insertion gain, defined as the difference between the insertion loss for the ultrasonically sculpted waveguide and external lens, and relative enhancement for scattering media with different optical depths. The blue circles represent the experimental data, while the red line shows the average gain and standard error at each optical depth. The sample size (number of blue circles) is three.

We have configured and built a custom experimental setup to compare experimental and simulation results (Fig. 7). We immersed a piezoelectric cylindrical transducer (Boston Piezo-Optics Inc.) in a transparent acrylic tank filled with deionized (DI) water (Fig. 7a). We plot the frequency-amplitude map for this transducer (Fig. 8a). A collimated laser light (OBIS LX 640 nm, Coherent Inc.) with a diameter of 370 μm passes through a 200 μm pinhole (P200D, Thorlabs, Inc.) and passes from the bottom of the tank, propagating through the center of the transducer’s cavity. The imaging system consists of a monochrome CMOS camera (CS505MU, Thorlabs, Inc.), a zoom lens (VZM 600i, Edmund Optics Inc.), and an optical window (WG11050-A, Thorlabs, Inc.). We immersed the optical window inside the DI water. The focal plane of the imaging system coincides with the top surface of the transducer. We have driven the transducer at 7.8 MHz (corresponding to its resonance frequency in water). To focus the light at the target region of interest, we have driven the ultrasonic transducer at an input electric potential of 7.5V for first focus and 33V for second focus (Fig. 7b, f). We have modulated the input laser ON-OFF at the frequency of ultrasound with a duty cycle of 10%, synchronized with the peak of ultrasound.

We first compare the experimental results and simulations in a transparent medium (DI water), as we show in Fig. 7. We visually observe a good match between experimental results (Fig. 7c) and simulations (Fig. 7d) at the first focal locus by comparing the spatial distribution of irradiance. We plot the radial cross-sections of the experimental and simulation results in Fig. 7e. We observe a reasonably good match between the simulation and experimental results. We can also observe a match between experiments and simulations for the second focus images (Fig. 7g, h) and their cross-sectional profiles (Fig. 7i).

One difference is the mismatch in the intensity around the center (inset-1 of Fig. 7i). We should note that the intensity profile predicted by the simulator is symmetric, as expected in theory, while the experimental data is slightly asymmetric. Intrinsic limitations of experimental conditions cause this mismatch. For example, the electric wires soldered on the transducer walls most likely contribute to altering the perfect cylindrical symmetry of the cavity.

Another difference is the intensity ripples introduced by diffraction effects (inset-2 of Fig. 7i), not modeled by the simulator. Diffraction causes intensity ripples due to constructive and destructive interference, while the simulated image shows the averaged version of such intensity ripples. As the light throughput refers to the flux integrated, the effect of these diffraction ripples averages out and do not affect our conclusions.

Next, we performed experiments to demonstrate that virtual optical waveguides can recycle and guide scattered light in scattering media. We used the first focus generated by the transducer; this is because the transducer we used to conduct experiments generates a more stable first focus than second focus. In these experiments, we confined a beam of light through 11 mm of scattering medium using a virtual optical waveguide, as represented in Fig. 7c, d. We placed an optical lens (AC127-019-A-ML, Thorlabs, Inc.) with a focal length of 19 mm in the air outside the tank. The external lens was chosen to focus light at the same target location (11 mm into the medium) with a comparable spot size (FWHM = 18 μm).

We use the insertion loss (4) and enhancement (6) defined in the previous subsection to quantify the relative improvement of the light throughput. Since the experimental conditions are the same for both transparent and scattering media, we expect that the difference in insertion loss is due to the difference in the portion of scattered light reaching the target plane. The increase in the number of scattered light for virtual optical waveguide is a result of the recycled scattered light.

We performed multiple experiments in transparent DI water and scattering media composed of different concentrations of intralipid 20% emulsion (I141, Sigma-Aldrich, USA) mixed in water. Using these different concentrations, we could perform experiments to compare the performance of the virtual optical waveguide and the external lens at different optical depths, ranging from 0.5TMFP to 2.5TMFP with a step size of 0.5TMFP. The anisotropy factor for most biological tissue is around 0.9, so the maximum measured optical depth would correspond to around 25MFP, which is approximately equal to 1.25 mm if the medium is skin tissue^29^ and 1.875 mm if the medium is brain tissue^30^. These values indicate the optical depth of the medium inside the transducer cavity, for a physical thickness of 11 mm, corresponding to the length of the transducer. In these experiments, there was also a 10 mm gap between the transducer and optical windows of the imaging system. Therefore, the light travels through an additional scattering medium before reaching the top imaging microscope. Considering this additional gap, the optical depth range of the medium would range from 0.95TMFP to 4.77TMFP.

For each optical depth, we repeated the experiment using three different batches of the scattering medium. For each batch, we captured the data three times to consider possible variabilities introduced by non-ideal experimental conditions, such as fluctuations in the laser power, oscillations in the signal driving the transducer, and potential microscopic bubbles or debris fluctuating within the field of view of the camera. We averaged the results for the experiments for each batch.

We have plotted the insertion gain in Fig. 8b, where we can observe a significant enhancement of light throughput up to 15%. We also observe an increasing trend indicating that the insertion gain increases with the optical depth of the medium. We have anticipated this trend as scattered light increases with the optical depth of the medium, and as a consequence, the effect of light recycling becomes more prominent. We also anticipate that this trend is monotonic but reaches a plateau and starts decreasing for larger optical depths, where the effect of scattering becomes dominated. The virtual waveguide cannot effectively guide and recycle light in-between successive scattering events. We could observe this expected outcome by the diminishing enhancement between 2 − 2.5TMFP in Fig. 8b.

## Discussion

We presented a rigorous analysis of the effect of ultrasonic parameters and optical properties on the light throughput achieved by using ultrasonically sculpted virtual optical waveguides to focus light inside a transparent or turbid medium. To perform our analysis, we developed a simulator for light propagation inside media with a sculpted virtual optical waveguide. Our simulator is physically accurate, accounting for both continuous refraction and scattering effects, and computationally efficient.

We used our simulator to thoroughly evaluate how light throughput varies as a function of ultrasonic parameters, in both transparent and scattering media. We performed additional simulations to compare using virtual optical waveguide versus an ideal external lens to focus light inside media with optical parameters mimicking those of human bladder tissue, taking into account constraints on ultrasonic parameters due to tissue safety limits. Our simulation-based analysis shows that the ultrasonic parameters maximizing light throughput can be very different in transparent versus turbid media, and that using a well-configured virtual optical waveguide can improve relative light throughput in human bladder tissue by up to 50%, compared to using an ideal external lens. These findings highlight the ability of virtual optical waveguides to both focus ballistic light and recycle scattered light. They also demonstrate the importance of accurately simulating both scattering and continuous refraction for analyzing virtual optical waveguides.

We validated the accuracy of our simulation-based analysis, by comparing simulation results with experiment measurements of light throughput in turbid media. Our experiments demonstrated for the first time that virtual optical waveguide effectively recycle scattered light, and can improve relative light throughput compared to an external lens by up to 15% at a focusing depth of 5TMFP.

Our analysis shows that using higher ultrasonic frequencies to sculpt virtual optical waveguides can lead to significant improvements in light throughput in turbid media, without violating tissue safety limits. However, achieving this improved performance in practice is currently difficult, because of the lack of commercial ultrasonic transducers that can operate at frequencies above ~8 MHz. Therefore, our analysis highlights the importance of developing ultrasonic transducer hardware that can operate at higher frequencies.

We note that a drawback of using higher ultrasonic frequencies is the increased ultrasound propagation loss. By increasing the ultrasound frequency from ~ 1 MHz in previous work^1^ to ours at 30.52 MHz, the propagation loss of ultrasound in biological tissue is increased by a factor of 7.68. This attenuation can be compensated by increasing the ultrasound power at the source. To prevent damage to the biological tissue, the ultrasound power at the source can be distributed over a larger area to minimize the local intensity, and then ultrasound can be focused to reach the target region of interest within tissue. High frequency ultrasound (e.g., at 40 MHz) has been used for biological applications^31, 32^.

Even though we focused on cylindrical transducers, with Bessel-shaped pressure profiles, our simulation algorithm and implementation can be used to analyze and configure virtual waveguides using other ultrasonic patterns, for example generated by traveling-wave or focused ultrasound transducers. Additionally, we analyzed virtual optical waveguide performance in only transmissive configurations, where the incident beam and the target region are at opposite sides of a medium. In the future, it would be interesting to use our algorithm to perform a similar analysis in reflective configurations, where the incident beam and the target region are at the same side of the medium.

## Methods

### Monte Carlo rendering simulator

Our simulator uses Monte Carlo rendering to produce estimates of radiometric measurements accurate under the assumptions of geometric optics. To achieve physical accuracy, our simulator accounts for two effects.

The first effect is the bending of light rays traveling through a medium with continuously-varying refractive index. A light ray starting at position **x** inside the medium with direction **ω** will evolve, in the absence of scattering, according to Hamilton’s equations^33^:

7 $$\frac{{{{{{{{\rm{d}}}}}}}}{{{{{{{\bf{v}}}}}}}}}{{{{{{{{\rm{d}}}}}}}}s}=\nabla n({{{{{{{\bf{x}}}}}}}}),\,\frac{{{{{{{{\rm{d}}}}}}}}{{{{{{{\bf{x}}}}}}}}}{{{{{{{{\rm{d}}}}}}}}s}=\frac{{{{{{{{\bf{v}}}}}}}}}{n({{{{{{{\bf{x}}}}}}}})},$$

where  d*s* is the infinitesimal arc length along the ray, *n*(**x**) is the refractive index at location **x** (given by Equation (1)), and **v** ≡ *n*(**x**)**ω** is the velocity of light. Hamilton’s equations can be evaluated numerically using symplectic integration techniques such as the leapfrog integrator^34^.

The second effect is the scattering of light traveling through a turbid medium. For media such as tissue, the radiance $L\left({{{{{{{\bf{x}}}}}}}},{{{{{{{\boldsymbol{\omega }}}}}}}}\right)$ at location **x** inside the medium towards direction **ω** will satisfy, in the absence of continuous refraction, the radiative transfer equation (RTE)^35^:

8 $${{{{{{{\boldsymbol{\omega }}}}}}}} \nabla {L}\left({{{{{{{\bf{x}}}}}}}},{{{{{{{\boldsymbol{\omega }}}}}}}}\right)=-{\sigma }_{t}L\left({{{{{{{\bf{x}}}}}}}},{{{{{{{\boldsymbol{\omega }}}}}}}}\right)\\+a{\sigma }_{t}{\int}_{{{\mathbb{S}}}^{2}}{f}_{s}\left({{{{{{{\boldsymbol{\omega }}}}}}}}\cdot {{{{{{{{\boldsymbol{\omega }}}}}}}}}^{{\prime} }\right)L\left({{{{{{{\bf{x}}}}}}}},{{{{{{{{\boldsymbol{\omega }}}}}}}}}^{{\prime} }\right)\,{{{{{{{\rm{d}}}}}}}}\sigma \left({{{{{{{{\boldsymbol{\omega }}}}}}}}}^{{\prime} }\right),$$

where ${{\mathbb{S}}}^{2},\,{{{{{{{\rm{d}}}}}}}}\sigma \left({{{{{{{{\boldsymbol{\omega }}}}}}}}}^{{\prime} }\right)$ are the unit sphere and associated area measure, respectively, and *σ*_*t*_, *a*, *f*_*s*_ are the extinction coefficient, albedo, and phase function, respectively, of the medium. The phase function *f*_*s*_ is typically assumed to be a Henyey-Greenstein phase function^16^ of anisotropy factor *g*. We note that it is common to describe the optical density of the medium using its mean free path MFP ≡ 1/*σ*_*t*_ (another common term to describe the optical density is scattering mean free path SMFP ≡ 1/*σ*_*s*_ and *σ*_*s*_ = *aσ*_*t*_.), and the optical depth of the medium in units of MFP (i.e., ratio of geometric depth and MFP). Alternatively, the literature also describes optical density in terms of the transport mean free path ${{{{{{{\rm{TMFP}}}}}}}}\equiv 1/{\sigma }_{t}\left(1-g\right)$, and correspondingly the optical depth in units of TMFP (i.e., ratio of geometric depth and TMFP). The RTE can be simulated accurately using Monte Carlo volumetric rendering techniques^36^.

In media where there is both continuous refraction and scattering, Eqs. (7) and (8) can be combined into the refractive radiative transfer equation (RRTE)^12^. Pediredla et al.^13^ developed the first unbiased Monte Carlo technique for simulating the RRTE, which they have shown can accurately reproduce experimental measurements from turbid media with sculpted virtual optical waveguides. Their simulator emphasizes generality, supporting arbitrary scenes. Unfortunately, this generality comes at the cost of reduced computational efficiency: They report that simulating a single virtual optical waveguide experiment can take up to 260 h. This makes using their simulator for our analysis, which requires simulating hundreds of such experiments, impractical. We overcome this obstacle by developing a simulator tailored to the configuration of Fig. 1, which is two orders of magnitude faster than the general-purpose simulator of Pediredla et al.^13^. Below we describe our simulator, starting from the case of temporally-constant refractive index values, then continuing with the temporally-varying case.

#### Algorithm 1

Monte Carlo rendering algorithm.



**Fig. 9:**
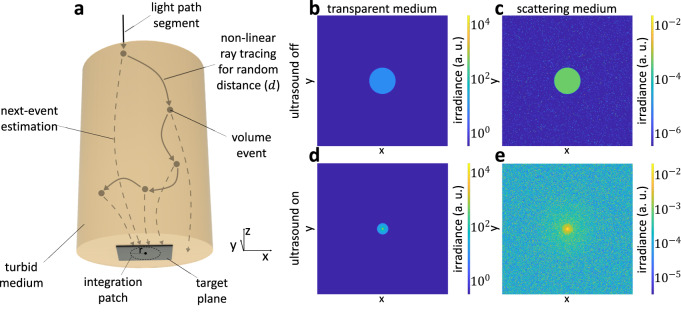
Monte Carlo rendering algorithm and sample renderings (simulation). **a** We use a variant of particle tracing with next-event estimation to simulate light propagation in turbid media with ultrasonically sculpted virtual optical waveguides. We show sample renderings for (**b**) ultrasound off and transparent medium (**c**) ultrasound off and turbid medium (10MFP) (**d**) ultrasound on and transparent medium (**e**) ultrasound on and turbid medium (10MFP). With ultrasound, we observe that both ballistic and scattered light is guided towards the center.

Temporally-constant refractive index. Our simulator uses a Monte Carlo rendering algorithm, visualized in Fig. 9a, that is a variant of volumetric particle tracing^37^. The Monte Carlo rendering algorithm estimates the total flux incident on the target area by aggregating radiance contributions from multiple stochastically-generated light paths that start at the incident beam and end at the target area. When generating a light path, our algorithm first initializes it to start at a randomly sampled location on the incident beam and to have the beam direction. The algorithm then traces the path by alternating between non-linear ray tracing—integrating Hamilton’s Eq. (7), to propagate the path for some randomly sampled distance—and volume events—randomly either terminating due to absorption, or changing its direction of propagation due to scattering. The algorithm continues the tracing procedure until the path reaches the target area, leaves the medium, or is absorbed. The algorithm samples random propagation distances, absorption events, and scattering directions based on the extinction coefficient, albedo, and phase function, respectively, of the turbid medium; and uses the refractive index of the virtual optical waveguide for propagation. The final flux estimate includes contributions from both ballistic and scattered light paths that are guided by the virtual optical waveguide between scattering events.

The above particle tracing procedure, though physically accurate, can be very inefficient, because most traced light paths will not reach the target area. To mitigate this problem, we modify our algorithm to use a form of next-event estimation^37^: At each volume event, our algorithm samples a direction pointing from the current point towards the target area, then uses non-linear ray tracing to propagate a sub-path. Even though this next-event estimation procedure does not guarantee that the sub-path will reach the target area, it greatly increases the probability of forming paths that contribute to the flux estimate. Empirically, we found that combining our particle tracing and next-event estimation procedures accelerates convergence by orders of magnitude compared to using just particle tracing, without introducing any bias. Using this combination, our specialized simulator is also faster than the general-purpose simulator of Pediredla et al.^13^ when applied to the scene reported by Pediredla et al.: for the quadrupole beam pattern formed in the scattering medium they report (depth = 30 mm, *σ*_*t*_ = 100 m^−1^, *g* = 0.85, frequency 813 kHz), we verified that our specialized simulator is ~240 times faster than their general-purpose simulator (runtime of 1.08 h for our simulator, versus a reported runtime of 260 h for their simulator), and that the two produce the same flux estimates.

Temporally-varying refractive index. The algorithm we described above assumes that the refractive index of the medium, though spatially-varying, is constant over time. However, the ultrasonically sculpted refractive index of Eq. (1) varies over time as a sinusoid, at the same frequency as the ultrasonic waves. We note that the temporal dynamics of ultrasound and light differ by several orders of magnitude. Given this, we can model the effects of the dynamic changes of refractive index by averaging flux estimates from multiple runs of our algorithm, with each run using temporally-constant refractive index values corresponding to a specific value of *t*. Equivalently, we modify our algorithm so that, for each path it generates, it first randomly samples a value of *t*, then traces the path using the refractive index values for that *t* from Eq. (1).

This modification concludes our algorithm, which we summarize in Algorithm 1. We emphasize that our algorithm accounts for both ballistic and scattered light, unlike previous simulators that can model only ballistic light^38^. As we show in results section, this capability is critical for accurate analysis of light throughput in turbid media. For reproducibility, we provide our implementation and the virtual optical waveguide configurations we simulate as supplementary material^39^.

Figure 9b–e visualizes irradiance (flux density) values at all points at the target area at the output of a virtual optical waveguide, rendered using our simulator. We can use our simulator to simulate both transparent (first column) and turbid (second column) media, without (first row) and with (second row) ultrasonic modulation. From the second row, we observe that the virtual optical waveguide can focus light in both transparent and turbid media. In Fig. 9e, the halo around the focus point is due to scattered light that is “recycled” and guided close to the region surrounding the focus point. This recycled scattered light can help increase light throughput in applications requiring focusing of light through turbid media.

### Reporting summary

Further information on research design is available in the Nature Portfolio Reporting Summary linked to this article.

### Supplementary information

Reporting Summary

## Data Availability

The data is available at 10.5281/zenodo.8118479.

## Code availability

The simulator and the code for processing data and generating various figures is available at 10.5281/zenodo.8118479.
